# Uracil Derivatives for Halogen-Bonded Cocrystals

**DOI:** 10.3390/ijms221910663

**Published:** 2021-10-01

**Authors:** Mónica Benito, Yannick Roselló, Miquel Barceló-Oliver, Antonio Frontera, Elies Molins

**Affiliations:** 1 Institut de Ciència de Materials de Barcelona (ICMAB-CSIC), Campus UAB, 08193 Bellaterra, Spain; 2 Departament de Química, Universitat de les Illes Balears, Ctra. Valldemosa km 7.5, 07122 Palma de Mallorca, Spain; ynkrm11@gmail.com (Y.R.); miquel.barcelo@uib.es (M.B.-O.)

**Keywords:** nucleobases, cocrystal, crystal engineering, mechanochemistry, noncovalent interactions, DFT

## Abstract

Among non-covalent interactions, halogen bonding is emerging as a new powerful tool for supramolecular self-assembly. Here, along with a green and effective method, we report three new halogen-bonded cocrystals containing uracil derivatives and 1,2,4,5-tetrafluoro-3,6-diiodobenzene as X-bond donor coformer. These multicomponent solids were prepared both by solvent-drop grinding and solution methods and further characterized by powder and single-crystal X-ray diffraction, Fourier-transformed infrared spectroscopy, and thermal methods (TGA-DSC). In order to study the relative importance of hydrogen versus halogen bonds in the crystal packing, computational methods were applied.

## 1. Introduction

In 2013, after several years working on the rationalization and unification of interactions involving halogens, the IUPAC defined the halogen bond as a non-covalent interaction when there is evidence of a net attractive interaction between an electrophilic region associated with a halogen atom in a molecular entity and a nucleophilic region in another, or the same, molecular entity [1].

Over the last decades, the use of this non-covalent interaction has grown rapidly as shown by several reviews [2,3,4,5]. A plethora of opportunities in areas such as organic chemistry [4], supramolecular chemistry [5], crystal engineering including pharmaceutical cocrystals [6,7,8,9,10,11], polymer science [12,13], medicinal chemistry, and chemical biology [14,15,16,17,18] have grown, taking advantage of its high linear directionality, tunable bonding strength, hydrophobicity, or the size of the halogen atom. Furthermore, in biological systems such as proteins or nucleic acids, halogen bonding contribution has been found in many protein-ligand complexes corroborated by several publications [19,20]. Particularly, in the case of nucleobases, some reported studies are theoretical [21,22], while others were carried out with halogenated nucleobases due to the use of these compounds as intermediate scaffolds for the synthesis of active pharmaceutical ingredients (APIs) [23,24]. However, alkylated nucleobases also represent important models for explaining the contribution of DNA methylation in gene expression through its regulation or inhibition [22]. For this reason, an increased effort from experimental and theoretical points of view is necessary for understanding this type of non-covalent interaction, in many cases with a similar strength to hydrogen bonds.

The renaissance of mechanochemistry has encompassed a whole range of possibilities not only for crystal engineering and polymorphism but also for synthetic processes, especially when products different from the ones obtained via solution methods are accomplished. From a green chemistry perspective, this synthetic method as well as other solvent-free and more sustainable approaches are highly demanded [25]. The required activation energy is obtained by mechanical forces in solid-state grinding methods. Moreover, although far away from solvent techniques, the addition of catalytic amounts of solvent, known as liquid-assisted grinding (LAG), has even permitted a great advance.

**Scheme 1:**
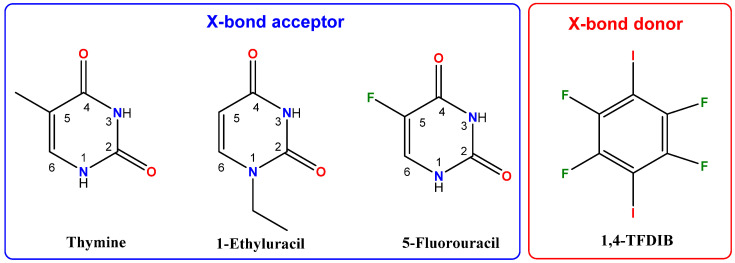
Uracil derivatives and the halogenated coformer used in this work.

In previous work, we reported the preparation using the mechanochemical method of a halogen bonded cocrystal based on 9-ethyladenine and 1,2,4,5-tetrafluoro-3,6-diiodobenzene (TFDIB) [26]. The present contribution is devoted to the ability of three derivatives of uracil for halogen bonding, taking advantage of this strategy. The selected compounds are the canonical nucleobase thymine (THY), the aliphatic derivative 1-ethyluracil (1ETURA), and the antineoplastic and well-known API 5-fluorouracil (5FU). All of them have been used as building blocks for preparing halogen-bonded cocrystals with the classical iodofluorobenzene compound (see Scheme 1) through the mechanochemical method. Furthermore, solution crystallizations to afford suitable single crystals for their structural resolution as well as the physico-chemical characterization of the solids were performed. Finally, DFT calculations of the three new cocrystals containing these uracil derivative molecules as X-bond acceptors and TFDIB as X-bond donor were conducted.

## 2. Results and Discussion

### 2.1. Spectroscopic Characterization

As stated before, from the supramolecular perspective, all the nucleobases show potential groups for forming non-covalent interactions such as hydrogen bonds or π–π stacking interactions. However, observation of other weak interactions such halogen bonds in the structural motifs could also be very interesting to know how nucleobase-based APIs interact. For this reason, an initial cocrystal screening using the mechanochemical method was carried out with canonical nucleobases as well as for two more derivatives: 1-ethyluracil and the active pharmaceutical ingredient 5-fluorouracil as target molecules. The coformer used was the ditopic and well-known 1,2,4,5-tetrafluoro-3,6-diiodobenzene. In our multicomponent screening, different stoichiometric compositions (nucleobase: coformer ratio: 1:1, 1:2, 2:1, and 4:1) and different solvents were explored.

**Figure 1:**
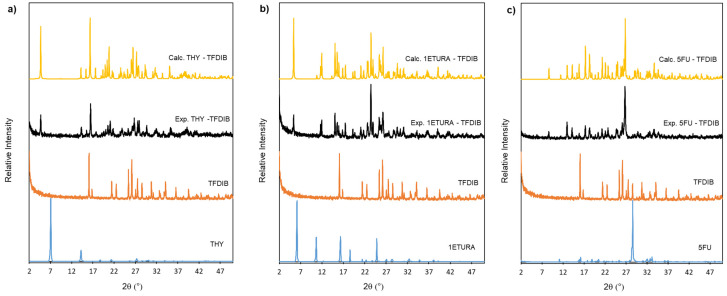
Comparison of calculated and experimental PXRD patterns for the cocrystals **1** (**a**), **2** (**b**), and **3** (**c**) and their respective precursors.

At first, the analysis by powder X-ray diffraction means of the solids after grinding confirmed the presence of new phases for the systems: thymine-TFDIB and 1ETURA-TFDIB in a nucleobase: coformer 2:1 molar ratio (Figure 1a,b), indicating that only the aliphatic substituted nucleobases, thymine or 1-ethyluracil, underwent cocrystal formation resulting in compounds **1** and **2**, respectively. The rest of the canonical nucleobases (adenine, guanine, uracil, and cytosine) remained unreacted as their powder patterns were the sum of the corresponding former substances even when different stoichiometric ratios or solvents were used during the grinding screening (Figures S1–S4). Nucleobases show poor aqueous solubilities and have high melting points (see Table S1). These are indicative of the strength of their homosynthon motifs and therefore their low affinity for rendering other multicomponent crystals. A CSD survey (version 2020.2.0, update August 2020) revealed that multicomponent solids of pure uracil or guanine nucleobases are scarce since one cocrystal was found for each one (VIFKUR [27] and QQQFGM [28]). In contrast, several multicomponent solids are well reported for adenine, cytosine, or thymine. Most of the solid forms described were salts for the first two and cocrystals for the last one. It is important to remark that thymine is the lowest melting point nucleobase as shown in Table S1.

Surprisingly, for 5-fluorouracil, a different powder pattern was obtained when using a mixture of precursors in 4:1 molar ratio. Figure 1c shows the resulting powder pattern which confirms the successful formation of the cocrystal by the excellent agreement with the simulated PXRD from the single crystal structure. A search in the CSD (version 2020.2.0, update August 2020) showed that this is the first halogen-bonded cocrystal with such a high stoichiometric ratio and the halogen bond interaction is between a carbonyl group as an acceptor and this ditopic coformer.

Refcode ZIJHAD reports the only example found for a halogen-bonded cocrystal in 4:1 ratio with a racemic sulfonamide and 1,4-DITFB containing a S=O···I interaction [29].

Finally, the measured PXRD patterns of the three cocrystals obtained by grinding are in good agreement with those calculated from single crystal data, thus confirming that all solids were obtained as pure (Figure 1).

Previous studies have proven that the halogen bonding formation provokes small shifts in the vibrational modes either for the donor or the acceptor X-bond bands. In this sense, a careful examination of the FTIR spectra of the new synthesized compounds and their parent compounds allowed us to check those differences. Typical bands for TFDIB appear at 1455, 937, and 755 cm^−1^ and correspond tentatively to the νC-C stretch ring, νC-F stretch, and νC-I asymmetrical stretch, respectively [30]. In the case of the compound **1** (THY-TFDIB), almost no changes were observed for the characteristic modes of the coformer, although differences in the carbonyl or the N-H and C-H regions were observed respective to thymine (Figure S5 and Table S2). For the new cocrystal **2** (1ETURA-TFDIB), a slight increase in the νC-I band was observed (758 cm^−1^) and in the region for C=O stretchings, some shifts were also observed (now at 1704 and 1651 cm^−1^) or again in the N-H and C-H region (3200–2800 cm^−1^) (Figure S6). Finally, the C=O and N-H stretchings in the 5-fluorouracil compound exhibited at 1720/1648 and 3115 cm^−1^, respectively. After formation of cocrystal **3**, the C=O band appeared at 1672 cm^−1^ and the N-H stretching band at 3150/3088 cm^−1^. Moreover, the three characteristic bands of the coformer suffered from a slightly increase and appeared at 1463, 943, and 758 cm^−1^ (see Figure S7). All these changes confirm the formation of new interactions in the synthesized compounds.

### 2.2. Thermal Analysis

TGA-DSC measurements were carried out to determine the formation of new crystalline solids. Moreover, by thermal analysis, the three compounds were confirmed to be anhydrous as no weight losses were observed before their melting (with decomposition). The TG (black curve) and DSC (orange curve) are displayed in the Supplementary Info. For compound **1** (THY- TFDIB), the melting was at approximately 139.7 °C (Tpeak), which is a temperature in between the melting of the precursors (see Table 1). During melting, decomposition of this cocrystal happened as indicated by a loss on drying (lod) of 60.3%, in agreement with the theoretical value for the release of a 1,2,4,5-tetrafluoro-3,6-diiodobenzene molecule (calc. 61%) (Figure S8) and also with the stoichiometric ratio confirmed by single-crystal resolution (see next section). In a similar manner, the TGA of compound **2** showed a lod of 59% (calc. 59% for one TFDIB molecule) during its melting process (T_p_: 154.7 °C) and this happened at a higher temperature than the corresponding ones for the precursors (1ETURA, T_p_ = 144.9 °C), showing a higher thermal stability (see Figure S9). Finally, in the TGA of compound **3**, the mass loss observed (42.7%) can also be attributed to the release of the coformer (calc. 43.6%). In the same range in the DSC trace, a single peak occurred at 161.8 °C, the melting of this cocrystal. A second peak at around 282.0 °C matches with melting of the 5-fluorouracil (Figure S10). This value was the highest for the new cocrystals and was in between the melting points of the former compounds (5-fluorouracil M_p_, 282–286 °C).

### 2.3. Crystal Structure Analysis

#### 2.3.1. Thymine–TFDIB (2:1) Cocrystal

**Figure 2:**
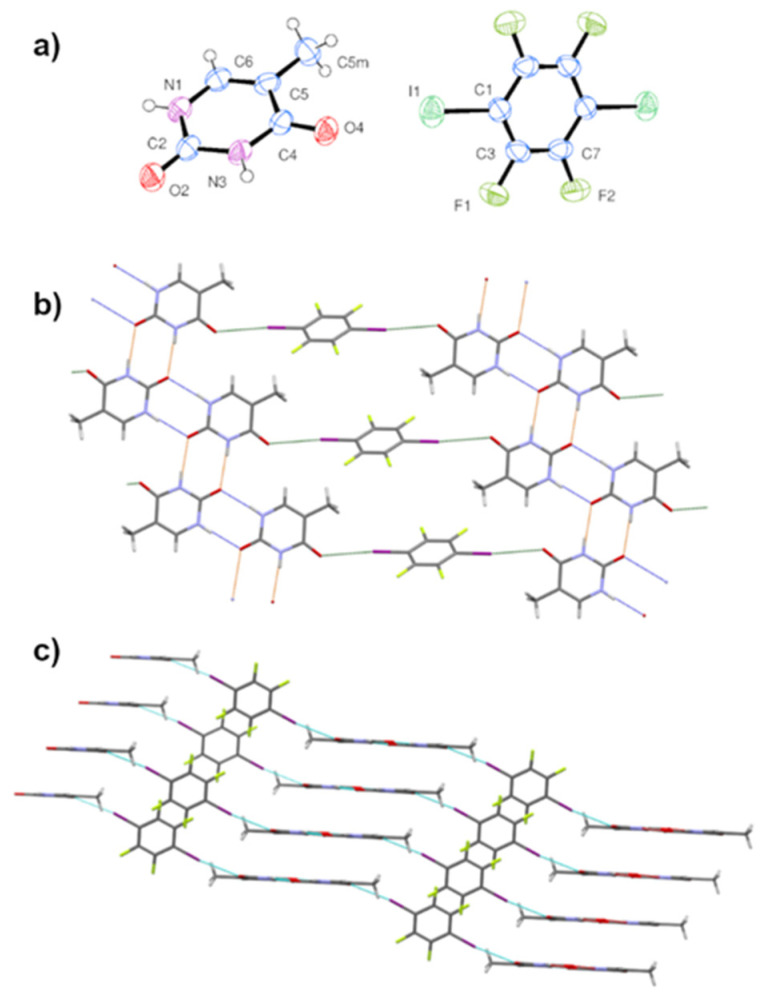
(**a**) ORTEP view of cocrystal **1** showing atom labeling. (**b**) 2D ladders formed by thymine–TFDIB molecules through H-bond and X-bond interactions. (**c**) 3D structure in cocrystal **1**.

Suitable single crystals of a thymine–TFDIB system for X-ray structural determination were grown from a mixture of methanol-acetone. Cocrystal **1** crystallizes in the triclinic space group *P*-1 containing two molecules of thymine and a molecule of the halogenated coformer in the asymmetric unit. No solvent molecules were found in agreement with the TGA trace. A representative ORTEP with atom labeling is shown in Figure 2a.

For this compound, each thymine molecule established four strong hydrogen bonds through N(1)-H···O(2) and N(3)-H···O(2) interactions by two distinct $R_{2}^{2}$(8) dimeric motifs and resulting in ribbons in a similar fashion as observed for polymorphic forms of thymine [31]. These ribbons were connected among them through symmetrically equivalent halogen bonds with the halogenated coformer through O(4)···I interactions (distance: 3.148 Å; angle: 172.26°) (Figure 2b). The resulting 2D ladders, in which the self-assembled nucleobases represent the steps of the ladder, were pilled forming the tridimensional structure shown in Figure 2c. Other short contacts were observed, such as π–π stacking among the nucleobases rings, through C(5M)-H···C(6) interactions or among the TFDIB rings by F(2)···C(3) contacts.

#### 2.3.2. 1-Ethyluracil–TFDIB (2:1) Cocrystal

**Figure 3:**
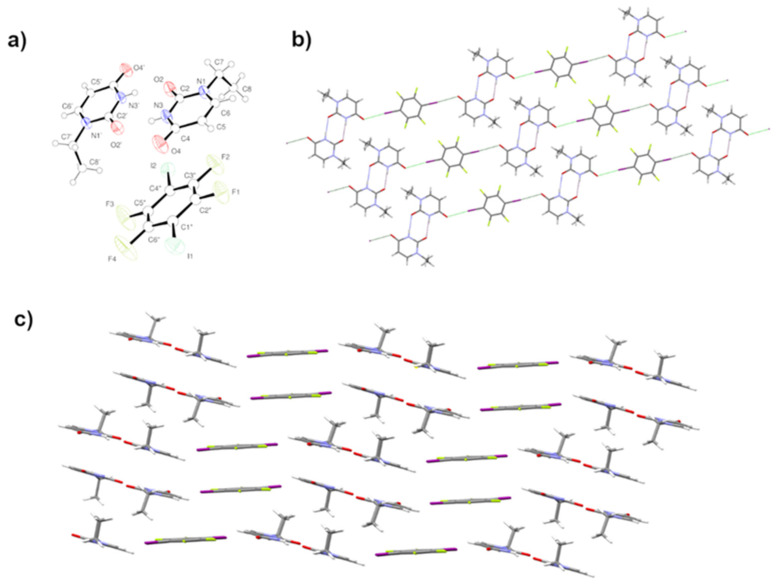
(**a**) ORTEP view of cocrystal **2** showing atom labeling in the asymmetric unit. (**b**) Planes formed by 1-ethyluracil–TFDIB molecules through H-bond and X-bond interactions. (**c**) 3D structure in cocrystal **2**.

This compound crystallized in the triclinic space group *P*-1 containing two molecules of 1-ethyluracil and one of 1,2,4,5-tetrafluoro-3,6-diiodobenzene in the asymmetric unit. No solvent molecules were present as confirmed by the thermogravimetric analysis. A representative ORTEP with atom labeling is shown in Figure 3a.

Unlike compound **1**, this structure was characterized by only one type of self-assembly between two molecules of 1-ethyluracil through a H-bond interaction among N(3)-H···O(2) and N(3′)-H···O(2) giving to $R_{2}^{2}$(8) motifs. The coformer served again as a bridge between two uracil dimers forming halogen bonding interactions through the O(4′)···I(1) or O(4)···I(2) (distances, 2.849 and 2.910 Å; angle: 175.25°) generating zig-zag chains (Figure 3b). Moreover, short contacts were formed between 1-ethyluracil molecules from different chains through C(7) or C(7′)-H from the ethyl group and O(4) or O(4′), C(6′)-H···F(1) and also through F···F contacts along the TFDIB molecules forming a 2D sheet. The final 3D structure was obtained by the stacking of these sheets in which the ethyl tails were faced every two sheets while the TFDIB or nucleobase rings were stacked with the next sheet, as displayed in Figure 3c.

#### 2.3.3. 5-Fluorouracil–TFDIB (4:1) Cocrystal

**Figure 4:**
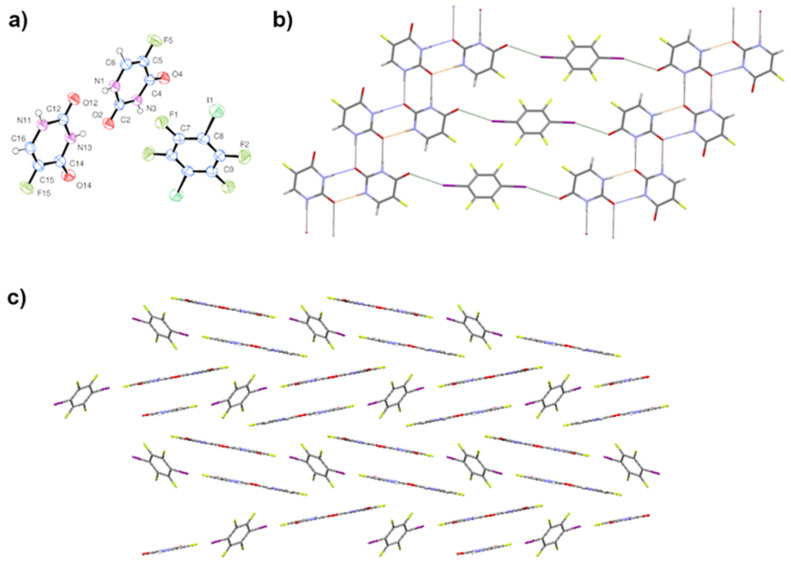
(**a**) ORTEP representation showing the cocrystal **3** with atom labeling. (**b**) Belts formed by 5-fluorouracil–TFDIB molecules through H-bond and X-bond interactions. (**c**) 3D structure in cocrystal **3**.

Compound **3** crystallized in the monoclinic space group *P*21/n containing two molecules of 5-fluorouracil and a half of 1,2,4,5-tetrafluoro-3,6-diiodobenzene in the asymmetric unit. No solvent molecules were present as confirmed by the thermogravimetric analysis. A representative ORTEP with atom labeling is shown in Figure 4a.

Self-assembly among the nucleobases was formed through bifurcated hydrogen bond interactions between O(2) and N(11) or N(13) and in a similar fashion, O(12) established bifurcated H-bonds with N(1) and N(3) forming zig-zag ribbons (see Figure 4b). As observed in the two previous structures, these ribbons were interconnected through the halogenated coformer which acted as a bridge forming bifurcated halogen bond interactions through O(4)···I(1) and F(5)···I(1) contacts, leading to belts in which the coformer was tilted from the plane formed by the nucleobases (dihedral angle between rings of TFDIB and 5FU: 61.4(6)°) and all the TFDIB rings were π–π stacked. The resulting belts were accommodated establishing further short contacts giving the final tridimensional structure shown in Figure 4c.

Interestingly, the presence of a methyl or a fluoro atom in C(5) in these uracil derivatives altered the hydrogen and halogen bond interactions observed in cocrystals **1** and **3**. This effect has also been observed previously when using hydroquinone as coformer with these two molecules (refcodes: OGIYOU and UDOVIV) [32,33].

The halogen bond interaction is typically colinear with an angle between the donor and acceptor components near to 175°. From the three cocrystals, **1** and **2** had angles of about 172.26 and 175.25°, respectively, in agreement with the described results in the literature. However, compound **3** was the one that had the lowest O···I-C angle, with a value of 160.03° as a result of a bifurcated X-bond also with the fluorine atom from the 5-fluorouracil (see for comparison Figure S11).

In our previous cocrystal with 9-ethyladenine and this coformer, N···I halogen bonds were obtained. It is interesting to remark that herein, the halogen bonds in the new cocrystals were formed through O···I similar to the multicomponent solids formed between the active pharmaceutical ingredient pentoxifylline with diiodotetrafluorobenzene or dibromotetrafluorobenzene [11]. However, while the ratio in the pentoxifylline–TFDIB cocrystal was 1:1 and halogen bond interactions among N(secondary nitrogen)···I or O(xanthyl fragment)···I were observed, for compounds **1** and **2**, the observed ratio was 2:1 and for cocrystal **3**, 4:1, and the halogen bonds were exclusively formed through O···I interactions. This behavior is closer to the cocrystal of pentoxifylline with the weaker halogen bond donor dibromotetrafluorobenzene. For multicomponent solids **1**–**3**, the available nitrogen atoms were involved in self-assembly between nucleobases.

Finally, the excellent agreement between the simulated powder patterns obtained from the crystal structures and the experimental from the bulk solids confirmed the results and the purity of new compounds (Figure 1).

### 2.4. Theoretical Study

The theoretical study was focused on the analysis of the energetic features of several supramolecular assemblies observed in the solid state of the cocrystals focusing on some recurrent motifs and comparing the energies H-bonding and halogen bonding interactions. This study is helpful to evaluate the relative importance of the interactions that govern the crystal packing.

**Figure 5:**
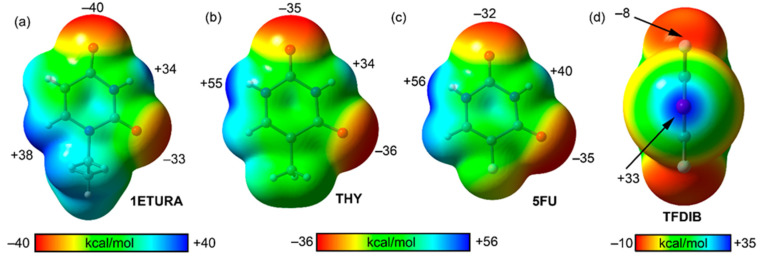
MEP surfaces of 1ETURA (**a**), THY (**b**), 5FU (**c**), and TFDIB (**d**) at the PBE0-D3/def2-TZVP level of theory. Isosurface 0.001 a.u. The values at selected points of the surface are given in kcal/mol.

Figure 5 shows the molecular electrostatic potential surfaces of all molecules used in this work. It can be observed that for the halogen bond acceptors (1ETURA, THY, and 5FU), the most negative atom was the O4-atom (ranging from −35 to −40 kcal/mol) followed by the O2 (ranging from −32 to −35 kcal/mol). The most positive values were located at the N1–H group (+55 kcal/mol in THY and +56 kcal/mol in 5FU). In 1ETURA, the presence of the ethyl group bonded to N1 caused changes in the charge distribution. In this case, the most positive region was located at the C–H group adjacent to N1 (+38 kcal/mol) followed by the N3–H group, which was slightly smaller (+34 kcal/mol). The MEP was more positive at the C–H group due to the contribution of the adjacent H-atoms belonging to the methylene group of the ethyl substituent. In THY and 5FU, the MEP at the N3–H group were +34 and +40, respectively, in line with the electron withdrawing effect of the fluorine substituent. For TFDIB coformer, the maximum MEP was located at the σ-hole of iodine (+33 kcal/mol) and the minimum at the F-atom (−8 kcal/mol). This MEP analysis anticipates that the uracil derivatives are strong H-bond donor and good H-bond and X-bond acceptors and that the coformer is a good X-bond donor.

**Figure 6:**
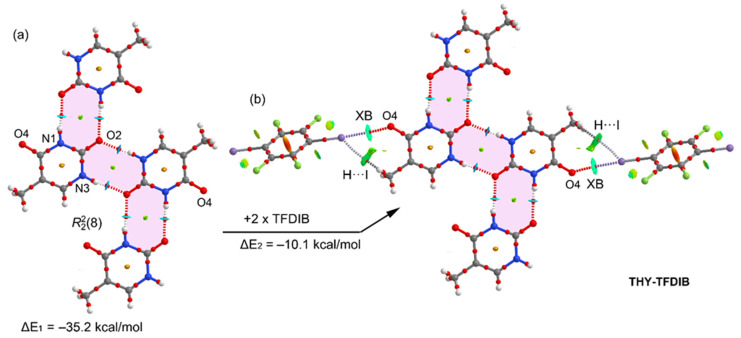
(**a**) QTAIM distribution of bond and ring CPs (red and yellow spheres, respectively) and NCIplot of the $R_{2}^{2}$(8) tetramer. (**b**) QTAIM distribution of bond and ring CPs (red and yellow spheres, respectively) and NCIplot of the hexamer of compound THY–TFDIB. The formation energies (ΔE) computed at the PBE0-D3/def2-TZVP level of theory are also indicated.

Figure 6a shows the two $R_{2}^{2}$(8) motifs observed in the solid state of THY–TFDIB with the participation of both NH groups as H-bond donors and the O2 atom as H-bond acceptor. The interaction energy of the tetrameric fragment used as model of the infinite 1D tape described above was ΔE_1_ = −35.2 kcal/mol, thus revealing the strong nature of the H-bonds (around −5.9 kcal/mol each H-bond). The strong nature of the H-bonds was confirmed by the NCIplot index analysis which revealed dark blue isosurfaces between the interacting atoms, coincident with the location of the bond critical points (CP, represented as a red sphere) that characterize the H-bonds. The formation of the self-assembled tetramer left the O4 atoms available as halogen bond acceptors. In fact, the two symmetrically equivalent and highly directional X-bonds that are shown in Figure 6b were evaluated by calculating the hexameric assembly, using the tetramer as starting material. The formation energy was ΔE_2_ = −10.1 kcal/mol, thus revealing that the X-bonds are slightly weaker than the H-bonds (around 5.0 kcal/mol). Each X-bond was characterized by a bond CP connecting the I to the O-atom and a blue isosurface located between the I and O-atoms (see Figure 6b). The color of the NCIplot isosurface was bluish, thus confirming that the X-bond is weaker than the H-bond, in agreement with the MEP analysis (the MEP value at the N1-H was significantly more positive than that at the σ-hole and the MEP value at N3–H was very similar). The NCIplot and QTAIM analysis also revealed the existence of a weak (green isosurface) contact between the H-atom of the thymine’s methyl group and the I-atom, characterized by a bond CP and bond path interconnecting both atoms.

**Figure 7:**
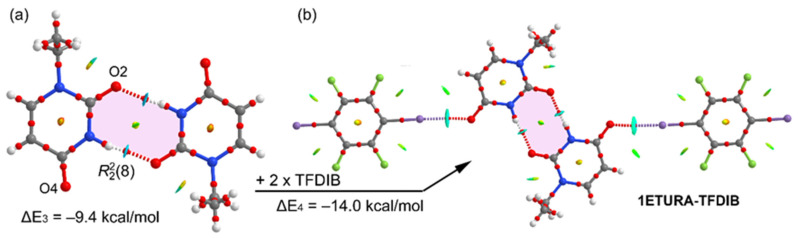
(**a**) QTAIM distribution of bond and ring CPs (red and yellow spheres, respectively) and NCIplot of the $R_{2}^{2}$(8) dimer of compound 1ETURA-TFDIB. (**b**) QTAIM distribution of bond and ring CPs (red and yellow spheres, respectively) and NCIplot of the tetramer of compound 1ETURA–TFDIB. The formation energies (ΔE) computed at the PBE0-D3/def2-TZVP level of theory are also indicated.

Figure 7a shows the $R_{2}^{2}$(8) motif observed in the solid state of 1ETURA–TFDIB. The H-bond acceptor was O2 thus leaving the most nucleophilic O4 atom available as halogen bond acceptor. The dimerization energy was ΔE_3_ = −9.4 kcal/mol, thus each HB was −4.7 kcal/mol, slightly smaller than the contribution of the H-bonds in the tetramer (formed by two different $R_{2}^{2}$(8) dimers) in compound THY-TFDIB in agreement with the MEP analysis (see Figure 5). Interestingly, the $R_{2}^{2}$(8) dimer established two symmetrically equivalent and highly directional X-bonds that were stronger than the H-bonds (ΔE_4_ = −14.0 kcal/mol, see Figure 7b) and also the X-bonds computed for the THY-TFDIB cocrystal. Each X-bond was characterized by a bond CP connecting the I to the O-atom and a blue isosurface located between the I and H-atoms (see Figure 7b).

**Figure 8:**
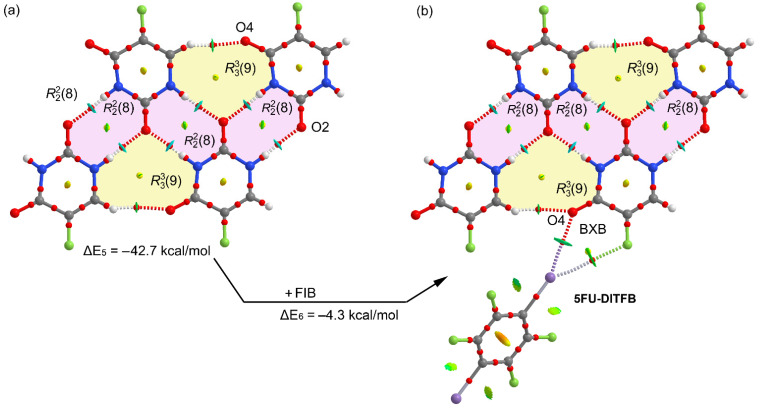
(**a**) QTAIM distribution of bond and ring CPs (red and yellow spheres, respectively) and NCIplot of the tetrameric assembly of compound 5FU-TFDIB. (**b**) QTAIM distribution of bond and ring CPs (red and yellow spheres, respectively) and NCIplot of the pentamer of compound 5FU-TFDIB. The formation energies (ΔE) computed at the PBE0-D3/def2-TZVP level of theory are also indicated.

Figure 8a shows the $R_{2}^{2}$(8) and $R_{3}^{3}$(9) motifs observed in the tetrameric assembly extracted from the solid state of 5FU-TFDIB showing the participation of both NH groups as H-bond donors and the O2 atom as H-bond acceptor. Moreover, the O4 established an H-bond with the C–H group of **5FU**, as evidenced by the bond CP and bond path connecting the CH to the O4-atom (see Figure 8a). In the solid state, both lone pairs of O2 participated in strong N–H···O H-bonds and one lone pair of O4 in the C–H···O H-bond, thus leaving one lone pair of the O4 atom available as halogen bond acceptor (see Figure 8b). The formation energy (ΔE_5_ = −42.7 kcal/mol) was larger (in absolute value) than the tetramer of compound 1 THY–TFDIB, likely due to the additional CH···O interactions. In this case, one 5FU unit of the $R_{3}^{3}$(9) synthon established a bifurcated X-bond (BXB) with one TFDIB molecule where both the O4 and F5 acted as electron donors. The interaction energy was quite modest (ΔE_6_ = −4.3 kcal/mol, see Figure 8b), thus suggesting that the bifurcated binding mode is weaker than the directional XBs observed in THY–TFDIB and 1ETURA–TFDIB co-crystals. The BXB was characterized by two bond CPs connecting the I to the O and F atoms of 5FU. The interaction was further characterized by a ring CP (yellow sphere) due to the formation of a supramolecular ring (see Figure 8b).

## 3. Materials and Methods

### 3.1. Materials

Thymine and 5-fluorouracil were obtained from commercial suppliers and used without further purification. 1-Ethyluracil was prepared following a modified synthesis reported previously [34]. TFDIB was purchased from Cymit Química S.L. (Barcelona, Spain). Methanol, acetone, acetonitrile, and ethyl acetate used for the synthesis and crystallization experiments were p.a. grade from Sigma-Aldrich (Merck KGaA, Darmstadt, Germany).

### 3.2. Methods

#### 3.2.1. Synthesis of the Cocrystal thymine–1,2,4,5-tetrafluoro-3,6-diiodobenzene (2:1)

A mixture containing thymine (100.08 mg, 0.79 mmol) and 1,2,4,5-tetrafluoro-3,6-diiodobenzene (159.49 mg, 0.40 mmol) in a 2:1 stoichiometric ratio was ground using a Retsch MM400 ball mixer (Retsch, Haan, Germany) in 10 mL agate grinding jars with two 5 mm agate balls at 30 Hz during 30 min.

Single crystals were obtained by slow evaporation of a solution containing a mixture of acetone and methanol.

#### 3.2.2. Synthesis of the Cocrystal 1-Ethyluracil–1,2,4,5-tetrafluoro-3,6-diiodobenzene (2:1)

A mixture containing 1-ethyluracil (75.19 mg, 0.54 mmol) and 1,2,4,5-tetrafluoro-3,6-diiodobenzene (107.74 mg, 0.27 mmol) in a 2:1 ratio was ground using a Retsch MM400 ball mixer (Retsch, Haan, Germany) in 10 mL agate grinding jars with two 5 mm agate balls at 30 Hz during 30 min.

Suitable single crystals were obtained by slow evaporation of methanol-ethyl acetate mixture.

#### 3.2.3. Synthesis of the Cocrystal 5-Fluorouracil–1,2,4,5-tetrafluoro-3,6-diiodobenzene (4:1)

A mixture containing 5-fluorouracil (75.0 mg, 0.58 mmol) and 1,2,4,5-tetrafluoro-3,6-diiodobenzene (57.9 mg, 0.144 mmol) in a 4:1 ratio was ground using a Retsch MM400 ball mixer (Retsch, Haan, Germany) in 10 mL agate grinding jars with two 5 mm agate balls at 25 Hz during 30 min.

Suitable single crystals were obtained by slow evaporation of a methanolic solution.

### 3.3. Characterization

Powder X-ray Diffraction (PXRD). PXRD data were collected using a Siemens D5000 powder X-ray diffractometer with Cu-Kα radiation (λ = 1.54056 Å), with 39 kV and 40 mA voltage and current applied. An amount of powder was gently pressed onto a glass slide to afford a flat surface and then analyzed. The samples were scanned from 2θ (°): 2–50 using a step size of 0.02° and a scan rate of 1 °/s.

Single Crystal X-ray Diffraction (SC-XRD). Suitable crystals of compounds **1**–**3** were selected for single crystal X-ray diffraction experiments and mounted at the tip of a nylon CryoLoop on a BRUKER APEX-II CCD diffractometer (Bruker, Karlsruhe, Germany) using graphite monochromated MoKα radiation (λ = 0.71073 Å). Crystallographic data were collected at 294(2) K. Data reduction was performed using SAINT V6.45A and SORTAV in the diffractometer package [35]. Data were corrected for Lorentz and polarization effects and for absorption by SADABS [36]. The structural resolution procedure was made using SHELXT [37]. Non-hydrogen atoms were refined anisotropically. Hydrogen atoms were introduced in calculated positions and refined riding on their parent atoms.

Calculated X-ray powder patterns were obtained from single crystal structure data using Mercury 4.3.1 software (CCDC, Cambridge, UK) [38].

In Table 2, details about general and crystallographic data for compounds **1**–**3** are summarized. Hydrogen-bond parameters can be found in Supplementary Information (Table S3).

Thermogravimetric analysis-Differential scanning calorimetry (TGA-DSC). Thermal analyses were carried out on a simultaneous thermogravimetric analysis (TGA)-differential scanning calorimetry/differential thermal analysis (heat flow DSC/DTA) system NETZSCH -STA 449 F1 Jupiter. Samples (3–8 mg) were placed in an alumina pan and measured at a scan speed of 10 °C min^−1^ from ambient temperature to 250 or 350 °C under N2 atmosphere as protective and purge gas (their respective flow velocities were 20 and 40 mL/min).

Attenuated Total Reflection Fourier Transform Infrared spectroscopy (ATR-FT-IR). Infrared spectra were recorded with a Jasco 4700LE spectrophotometer (JASCO, Tokyo, Japan) with attenuated total reflectance accessory. The scanning range was 4000 to 400 cm^−1^ and at a resolution of 4.0 cm^−1^.

### 3.4. Computational Methods

The theoretical study reported herein was carried out using the Gaussian-16 [39] program package (Gaussian, Wallingford, CT, USA) and the PBE0-D3/def2-TZVP level of theory [40]. Since we intend to estimate the interactions in the solid state, the crystallographic coordinates were used. This level of theory and methodology has been previously used to evaluate similar interactions [41]. In this work, the binding energy and interaction energy terms were used indistinctly. They were computed as the difference between the energies of the isolated monomers and their assembly and were corrected for the basis set superposition error (BSSE) by using the Boys–Bernardi counterpoise method [42]. The Bader’s quantum theory of “Atoms in molecules” (QTAIM) and the noncovalent interaction index (NCIPlot) were used to characterize the noncovalent interactions using the AIMall program [43]. The molecular electrostatic potential (MEP) surfaces were generated using the Gaussian-16 software (Gaussian, Wallingford, CT, USA) and the PBE0-D3/def2-TZVP wave-functions. The 0.001 a.u. isosurface was used as the best estimate of the van der Waals surface.

## 4. Conclusions

In conclusion, we prepared and characterized three new halogen-bonded cocrystals comprising the canonical nucleobase thymine and two derivatives, 1-ethyluracil and 5-fluorouracil, with the strong halogen-bond donor 1,2,4,5-tetrafluoro-1,3-diiodobenzene. All compounds form self-assembled H-bonded $R_{2}^{2}$(8) dimers in the solid state, which is an important and recurrent binding motif. The interactions were studied through DFT calculations and characterized by a combination of NCIPlot/QTAIM methods. In general, the behavior of the cocrystals reported herein agrees well with the MEP surface analysis. The energetic results reveal that the H-bonds are stronger than the halogen bonds apart from the 1ETURA–TFDIB cocrystal, where the X-bonds are stronger, likely due to the presence of the ethyl substituent in N1, which is the best H-bond donor group in THY and 5FU compounds. In view of the present results, our future perspective is to continue exploring the ability and importance for hydrogen and halogen bonds in other nucleobase-derivate APIs, as well as to determine the modification of their physico-chemical properties (solubility, stability, hygroscopicity, pharmacological activity). This type of study is scarcely found in the literature. Consequently, further investigation related to the formation of cocrystals is needed, particularly considering that many APIs contain halogen atoms in their structure and that cocrystals are a real alternative to salts and polymorphs in drug formulation.

## Figures and Tables

**Table 1:** Physical data of the starting compounds and the new cocrystals.

Compound	Mp (°C) *	ν_C=O_ (cm^−1^)	Cocrystal	Mp (°C)	ν_C=O_ (cm^−1^)	dO···I (Å)	ΔE (kcal/mol)
1,4-TFDIB	108–110	-	-	-	-	-	-
THY	320	1720/1667	**1**	139.7	1717	3.15	−10.1
1ETURA	144.9	1707/1645	**2**	154.7	1704	2.849; 2.910	−14.0
5FU	282–286	1720/1648	**3**	161.8	1710; 1672	3.097; 3.575	−4.3

* From literature.

**Table 2:** Crystallographic data and refinement for cocrystals **1**–**3**.

	1	2	3
Empirical Formula	C_16_H_12_F_4_I_2_N_4_O_4_	C_18_H_16_F_4_I_2_N_4_	C_22_H_12_F_8_I_2_N_8_O_8_
*M*r	654.11	682.15	922.20
Crystal system	Triclinic	Triclinic	Monoclinic
Space group	*P*-1	*P*-1	*P* 2_1_/n
a/Å	4.6812(15)	8.2094(7)	6.837(3)
b/Å	5.7924(19)	8.4322(7)	15.423(6)
c/Å	18.787(6)	16.4377(13)	13.611(5)
α/°	86.472(6)	87.684(2)	90
β/°	86.409(6)	87.889(2)	92.662(8)
γ/°	84.697(5)	83.752(2)	90
V/Å^3^	505.4(3)	1129.59(16)	1433.7(9)
Z	1	2	2
Radiation type	Mo K_α_	Mo K_α_	Mo K_α_
μ/mm^−1^	3.179	2.849	2.309
Temperature/K	294(2)	294(2)	294(2)
Crystal size/mm	0.27 × 0.26 × 0.15	0.330 × 0.130 × 0.100	0.340 × 0.080 × 0.080
D_calc_/g·cm^−3^	2.149	2.006	2.136
Reflections collected	10,285	33,259	31,225
Independent Reflections	2601 [R(int) = 0.0448]	5642 [R(int) = 0.0235]	3621 [R(int) = 0.0450]
Completeness to θ = 25.242°	99.8%	100.0%	99.7%
F(000)	310	652	884
Data/restraints/parameters	2601/0/137	5642/0/291	3621/0/241
Goodness-of-fit	1.277	1.039	1.065
Final R indices [I < 2d(I)]	R1 = 0.0607, wR2 = 0.1384	R1 = 0.0240, wR2 = 0.0573	R1 = 0.0434, wR2 = 0.0917
R indices (all data)	R1 = 0.0649, wR2 = 0.14	R1 = 0.0280, wR2 = 0.0599	R1 = 0.0563, wR2 = 0.0981
Largest diff. peak and hole/eÅ^−3^	1.505 and −1.394	0.847 and −0.551	1.468 and −1.280
CCDC n°	2,101,095	2,101,094	2,101,096

## Data Availability

Not applicable.

## Supplementary Materials

The following are available online at https://www.mdpi.com/article/10.3390/ijms221910663/s1, Figure S1. Comparison of the powder patterns of adenine (ADE), 1,2,4,5-tetrafluoro-1,3-diiodobenzene (TFDIB) and the mixtures prepared in methanol (MT) or water (WA) and the different ratios used. Figure S2. Comparison of the powder patterns of guanine (GUA), 1,2,4,5-tetrafluoro-1,3-diiodobenzene (TFDIB) and the mixtures prepared in methanol (MT) or water (WA) and the different ratios used. Figure S3. Comparison of the powder patterns of uracil (URA), 1,2,4,5-tetrafluoro-1,3-diiodobenzene (TFDIB) and the mixtures prepared in methanol (MT) or water (WA) and the different ratios used. Figure S4. Comparison of the powder patterns of cytosine (CYT), 1,2,4,5-tetrafluoro-1,3-diiodobenzene (TFDIB) and the mixtures prepared in methanol (MT), water (WA) or nitromethane (NM) and the different ratios used. Table S1. Melting points and solubilities in water of canonical nucleobases. Figure S5. Comparison of ATR-FT-IR spectra of thymine, 1,2,4,5-tetrafluoro-1,3-diiodobenzene (TFDIB) and cocrystal 1. Figure S6. Comparison of ATR-FT-IR spectra of 1-ethyluracil, 1,2,4,5-tetrafluoro-1,3-diiodobenzene (TFDIB) and cocrystal 2. Figure S7. Comparison of ATR-FT-IR spectra of 5-fluorouracil, 1,2,4,5-tetrafluoro-1,3-diiodobenzene (TFDIB) and cocrystal 3. Table S2. Some characteristic frequencies for the modified uracil compounds, the coformer and the new cocrystals. Figure S8. TGA-DSC of the cocrystal 1. Figure S9. TGA-DSC of the cocrystal 2. Figure S10. TGA-DSC of the cocrystal 3. Figure S11. X-bonds angles in cocrystals 1-3. Table S3. Hydrogen bonds for cocrystals 1 (THY-TFDIB), 2 (1ETURA-TFDIB) and 2 (5FU-TFDIB) [Å and °].

## References

[1] Desiraju G.R., Ho P.S., Kloo L., Legon A.C., Marquardt R., Metrangolo P., Politzer P., Resnati G., Rissanen K. (2013). Definition of the halogen bond (IUPAC Recommendations 2013). Pure Appl. Chem..

[2] Cavallo G., Metrangolo P., Milani R., Pilati T., Priimagi A., Resnati G., Terraneo G. (2016). The Halogen Bond. Chem. Rev..

[3] Costa P.J. (2017). The halogen bond: Nature and applications. Phys. Sci. Rev..

[4] Bulfield D., Huber S.M. (2016). Halogen Bonding in Organic Synthesis and Organocatalysis. Chem. Eur. J..

[5] Gilday L.C., Robinson S., Barendt T., Langton M.J., Mullaney B.R., Beer P.D. (2015). Halogen bonding in supramolecular chemistry. Chem. Rev..

[6] Christopherson J.-C., Topic F., Barret C.J., Friscic T. (2018). Halogen-bonded cocrystals as optical materials: Next-generation control over light–matter interactions. Cryst. Growth Des..

[7] Baldrighi M., Cavallo G., Chierotti M.R., Gobetto R., Metrangolo P., Pilati T., Resnati G., Terraneo G. (2013). Halogen bonding and pharmaceutical cocrystals: The case of a widely used preservative. Mol. Pharm..

[8] Baldrighi M., Bartesaghi D., Cavallo G., Chierotti M.R., Gobetto R., Metrangolo P., Pilati T., Resnati G., Terraneo G. (2014). Polymorphs and co-crystals of haloprogin: An antifungal agent. CrystEngComm.

[9] Aakeröy C.B., Welideniya D., Desper J., Moore C. (2014). Halogen-bond driven co-crystallization of potential anti-cancer compounds: A structural study. CrystEngComm.

[10] Miroslaw B., Plech T., Wujec M. (2015). Halogen bonding in the antibacterial 1,2,4-triazole-3-thione derivative—Spectroscopic properties, crystal structure. J. Mol. Struct..

[11] Choquesillo-Lazarte D., Nemec V., Cinčić D. (2017). Halogen bonded cocrystals of active pharmaceutical ingredients: Pyrazinamide, lidocaine and pentoxifylline in combination with haloperfluorinated compounds. CrystEngComm.

[12] Berger G., Soubhye J., Meyer F. (2015). Halogen bonding in polymer science: From crystal engineering to functional supramolecular polymers and materials. Polym. Chem..

[13] Berger G., Frangville P., Meyer F. (2020). Halogen bonding for molecular recognition: New developments in materials and biological sciences. Chem. Commun..

[14] Auffinger P., Hays F.A., Westhof E., Ho P. (2004). Halogen bonds in biological molecules. Proc. Natl. Acad. Sci. USA.

[15] Persch D.E., Dumele O., Diederich F. (2015). Molecular recognition in chemical and biological systems. Angew. Chem. Int. Ed..

[16] Wilcken R., Zimmermann M.O., Lange A., Joerger A.C., Boeckler F.M. (2013). Principles and applications of halogen bonding in medicinal chemistry and chemical biology. J. Med. Chem..

[17] Lu Y., Wang Y., Zhu W. (2010). Nonbonding interactions of organic halogens in biological systems: Implications for drug discovery and biomolecular design. Phys. Chem. Chem. Phys..

[18] Rowe R.K., Ho S. (2017). Relationships between hydrogen bonds and halogen bonds in biological systems. Acta Cryst..

[19] Kolář M.H., Tabarrini O. (2017). Halogen bonding in nucleic acid complexes. J. Med. Chem..

[20] Hardegger L.A., Kuhn B., Spinnler B., Anselm L., Ecabert R., Stihle M., Gsell B., Thoma R., Diez J., Benz J. (2011). Systematic Investigation of Halogen Bonding in Protein–Ligand Interactions. Angew. Chem. Int. Ed..

[21] Marín-Luna M., Alkorta I., Elguero J. (2015). The influence of intermolecular halogen bonds on the tautomerism of nucleobases. I. Guanine. Tetrahedron.

[22] Marín-Luna M., Alkorta I., Elguero J. (2016). The effect of cytosine methylation on its halogen-bonding properties. Comput. Theor. Chem..

[23] Valkonen A., Chukhlieb M., Moilanen J.O., Tuononen H.M., Rissanen K. (2013). Halogen and Hydrogen bonded complexes of 5-iodouracil. Cryst. Growth Des..

[24] Gerhardt V., Egert E. (2015). Cocrystals of 6-chlorouracil and 6-chloro-3-methyluracil: Exploring their hydrogen-bond-based synthon motifs with several triazine and pyrimidine derivatives. Acta Crystallogr. Sect. B Struct. Sci. Cryst. Eng. Mater..

[25] Howard J.L., Cao Q., Browne D.L. (2018). Mechanochemistry as an emerging tool for molecular synthesis: What can it offer?. Chem. Sci..

[26] Roselló Y., Benito M., Molins E., Barceló-Oliver M., Frontera A. (2019). Adenine as a halogen bond acceptor: A combined experimental and DFT study. Crystals.

[27] Thomas R., Kulkarni G. (2007). A hydrogen-bonded channel structure formed by a complex of uracil and melamine. Beilstein, J. Org. Chem..

[28] Madden J.J. (1973). The unit cell of a mixed crystal of guanine and 8-azaguanine. Acta Crystallogr. Sect. B Struct. Crystallogr. Cryst. Chem..

[29] Eccles K.S., Morrison R.E., Daly C.A., O’Mahony G.E., Maguire A., Lawrence S. (2013). Co-crystallisation through halogen bonding with racemic or enantiopure sulfinamides. CrystEngComm.

[30] Wang H., Jin W.J. (2017). Cocrystal assembled by 1,4-diiodotetrafluorobenzene and phenothiazine based on C-I···p/N/S halogen bond and other assisted interactions. Acta Cryst. Sect. B.

[31] Braun D.E., Gelbrich T., Wurst K., Griesser U.J. (2016). Computational and Experimental Characterization of Five Crystal Forms of Thymine: Packing Polymorphism, Polytypism/Disorder, and Stoichiometric 0.8-Hydrate. Cryst. Growth Des..

[32] Sridhar B., Babu Nanubolu J., Ravikumar K. (2015). Four cocrystals of thymine with phenolic coformers: Influence of the conformer on hydrogen bonding. Acta Cryst..

[33] Kumar S.S., Athimoolam S., Sridhar B. (2018). Structural, spectral, theoretical and anticancer studies on new co-crystal of the drug 5-fluorouracil. J. Mol. Struct..

[34] Roselló Y., Benito M., Barceló-Oliver M., Frontera A., Molins E. (2021). 1-Ethyluracil, a New Scaffold for Preparing Multicomponent Forms: Synthesis, Characterization, and Computational Studies. Crystallogr. Growth Des..

[35] Blessing R.H. (1995). An empirical correction for absorption anisotropy. Acta Crystallogr. Sect. A Found. Crystallogr..

[36] Bruker A.P.E.X., Saint A.X.S. (2008). Inc., Madison, WI, 2004 Search PubMed; (b) GM Sheldrick. 2004. Acta Crystallogr. Sect. A Fundam. Crystallogr..

[37] Sheldrick G.M. (2015). SHELXT—Integrated space-group and crystal-structure determination. Acta Crystallogr. Sect. A Found. Adv..

[38] Macrae C.F., Sovago I., Cottrell S.J., Galek P.T.A., McCabe P., Pidcock E., Platings M., Shields G.P., Stevens J.S., Towler M. (2020). Mercury 4.0: From visualization to analysis, design and prediction. J. Appl. Crystallogr..

[39] Frisch M.J.E.A., Trucks G.W., Schlegel H.B., Scuseria G.E., Robb M.A., Cheeseman J.R., Scalmani G., Barone V., Mennucci B., Petersson G. (2016). Gaussian 09, Revision d. 01, Gaussian.

[40] Adamo C., Barone V. (1999). Toward reliable density functional methods without adjustable parameters: The PBE0 model. J. Chem. Phys..

[41] Rozhkov A., Eliseeva A.A., Baykov S.V., Galmés B., Frontera A., Kukushkin V.Y. (2020). One-Pot Route to X-perfluoroarenes (X = Br, I) Based on FeIII-Assisted C–F Functionalization and utilization of these arenes as building blocks for crystal engineering involving halogen bonding. Cryst. Growth Des..

[42] Boys S.F., Bernardi F. (1970). The calculation of small molecular interactions by the differences of separate total energies. Some procedures with reduced errors. Mol. Phys..

[43] Keith T.A. (2013). AIMAll (Version 13.05.06).

