# Supplementary material for: Uracil Derivatives for Halogen-Bonded Cocrystals
Source: Int J Mol Sci. 2021 Oct 1;22(19):10663. doi: 10.3390/ijms221910663 (PMC8508742; doi:10.3390/ijms221910663)
Supplement: Supplementary file 1 [file ijms-22-10663-s001.zip › ijms-1392364-supplementary.pdf]

Supplementary Information

Uracil Derivatives for Halogen-bonded Cocrystals

Mónica Benito[1,*], Yannick Roselló[2,†], Miquel Barceló-Oliver[2], Antonio Frontera[2,*] and Elies Molins[1,*]

[1]Institut de Ciència de Materials de Barcelona (ICMAB-CSIC), Campus UAB, 08193 Bellaterra, Spain; mbenito@icmab.es (M.B.); elies.molins@icmab.es (E.M.)
[2]Departament de Química, Universitat de les Illes Balears, Ctra. Valldemosa km 7.5, E-07122 Palma de Mallorca, Spain; ynkrm11@gmail.com (Y. R.); miquel.barcelo@uib.es (M.B.-O.); toni.frontera@uib.es (A. F.)
†Present address: Department de Química Física i Inorgànica, Universitat Rovira i Virgili, 43007, Tarragona, Spain; yannick.rosello@urv.cat (Y.R.)

Content

Figure S1. Comparison of the powder patterns of adenine (ADE), 1,2,4,5-tetrafluoro-1,3-diiodobenzene (TFDIB) and the mixtures prepared in methanol (MT) or water (WA) and the different ratios used.

Figure S2. Comparison of the powder patterns of guanine (GUA), 1,2,4,5-tetrafluoro-1,3-diiodobenzene (TFDIB) and the mixtures prepared in methanol (MT) or water (WA) and the different ratios used.

Figure S3. Comparison of the powder patterns of uracil (URA), 1,2,4,5-tetrafluoro-1,3-diiodobenzene (TFDIB) and the mixtures prepared in methanol (MT) or water (WA) and the different ratios used.

Figure S4. Comparison of the powder patterns of cytosine (CYT), 1,2,4,5-tetrafluoro-1,3-diiodobenzene (TFDIB) and the mixtures prepared in methanol (MT), water (WA) or nitromethane (NM) and the different ratios used.

Table S1. Melting points and solubilities in water of canonical nucleobases.

Figure S5. Comparison of ATR-FT-IR spectra of thymine, 1,2,4,5-tetrafluoro-1,3-diiodobenzene (TFDIB) and cocrystal **1**.

Figure S6. Comparison of ATR-FT-IR spectra of 1-ethyluracil, 1,2,4,5-tetrafluoro-1,3-diiodobenzene (TFDIB) and cocrystal **2**.

Figure S7. Comparison of ATR-FT-IR spectra of 5-fluorouracil, 1,2,4,5-tetrafluoro-1,3-diiodobenzene (TFDIB) and cocrystal **3**.

Table S2. Some characteristic frequencies for the modified uracil compounds, the coformer and the new cocrystals.

Figure S8. TGA-DSC of the cocrystal **1**.

Figure S9. TGA-DSC of the cocrystal **2**.

Figure S10. TGA-DSC of the cocrystal **3**.

Figure S11. X-bonds angles in cocrystals **1**-**3**.

Table S3. Hydrogen bonds for cocrystals **1** (THY-TFDIB), **2** (1ETURA·TFDIB) and **3** (5FU·TFDIB) [Å and °].

Figure S1. Comparison of the powder patterns of adenine (ADE), 1,2,4,5-tetrafluoro-1,3-diiodobenzene (TFDIB) and the mixtures prepared in methanol (MT) or water (WA) and the different ratios used.

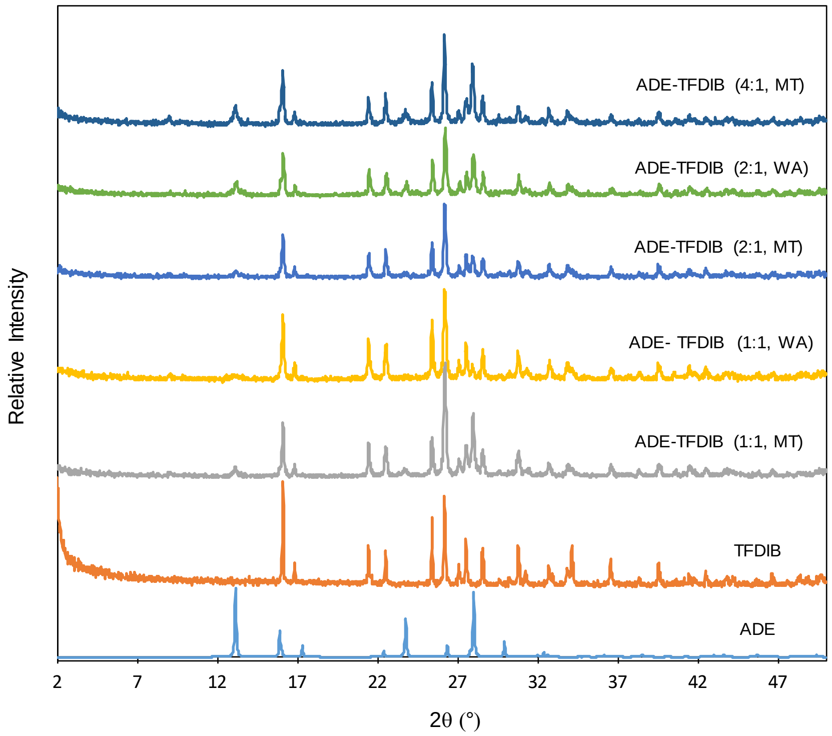

Figure S2. Comparison of the powder patterns of guanine (GUA), 1,2,4,5-tetrafluoro-1,3-diiodobenzene (TFDIB) and the mixtures prepared in methanol (MT) or water (WA) and the different ratios used.

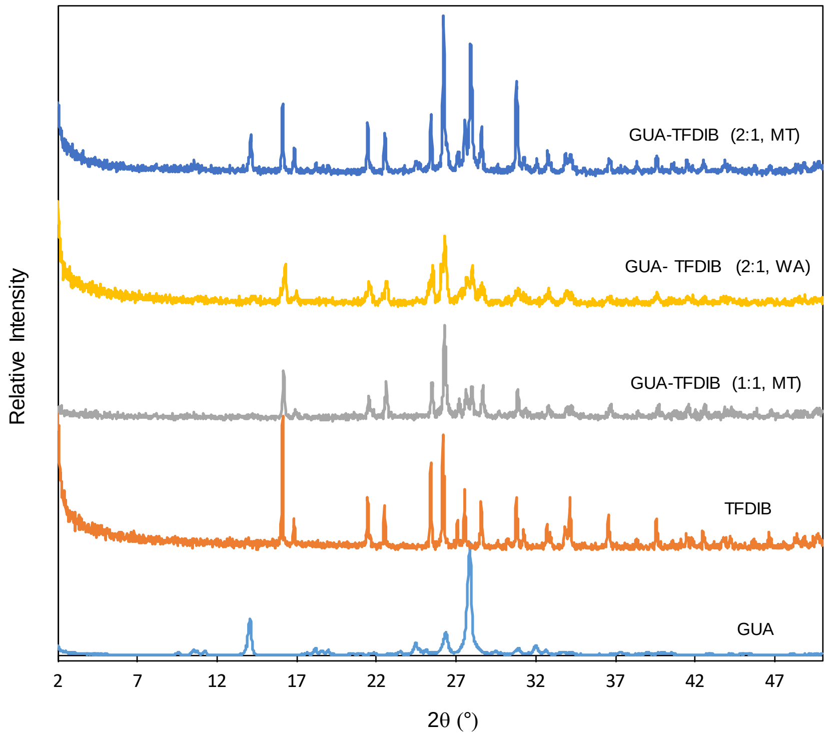

Figure S3. Comparison of the powder patterns of uracil (URA), 1,2,4,5-tetrafluoro-1,3-diiodobenzene (TFDIB) and the mixtures prepared in methanol (MT) or water (WA) and the different ratios used.

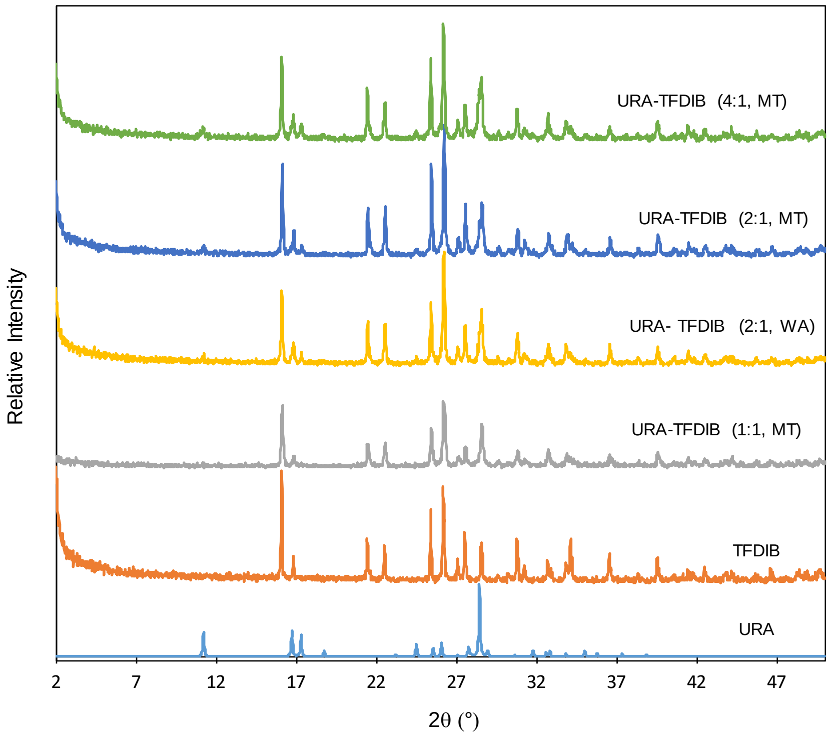

Figure S4. Comparison of the powder patterns of cytosine (CYT), 1,2,4,5-tetrafluoro-1,3-diiodobenzene (TFDIB) and the mixtures prepared in methanol (MT), water (WA) or nitromethane (NM) and the different ratios used.

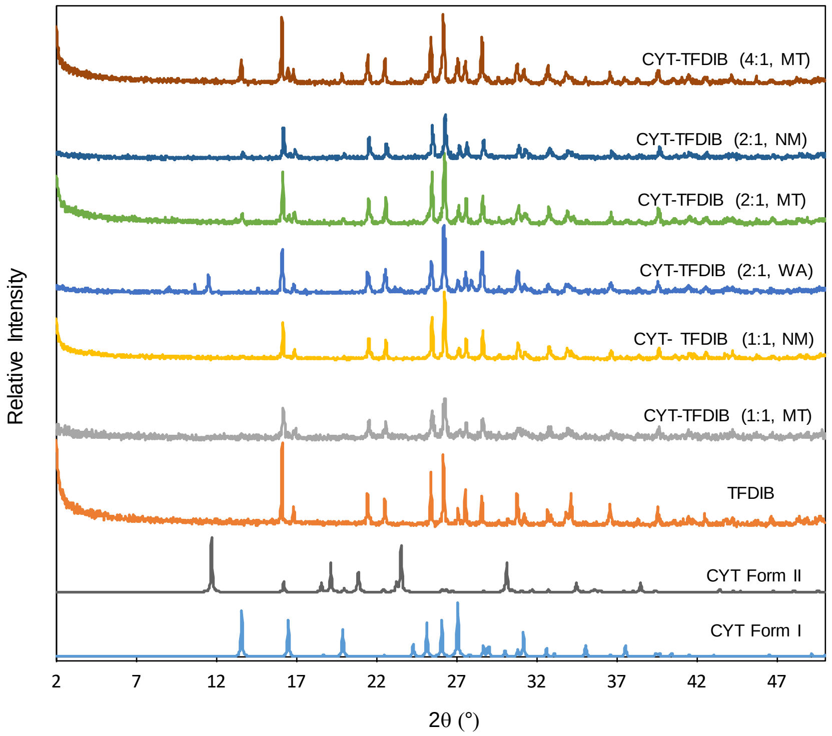

Table S1. Melting point and solubilities in water of canonical nucleobases.

Nucleobase	Mp (°C)	Solubility in water*
Adenine	360	1030 mg/L (25 °C)
Guanine	360	2080 mg/L (37 °C)
Uracil	338	3600 mg/L (25 °C)
Cytosine	320-325	8 mg/mL (25 ºC)
Thymine	316	3820 mg/L (25 °C)

*DrugBank website.

Figure S5. Comparison of ATR-FT-IR spectra of thymine, 1,2,4,5-tetrafluoro-3,6-diiodobenzene (TFDIB) and cocrystal **1**.

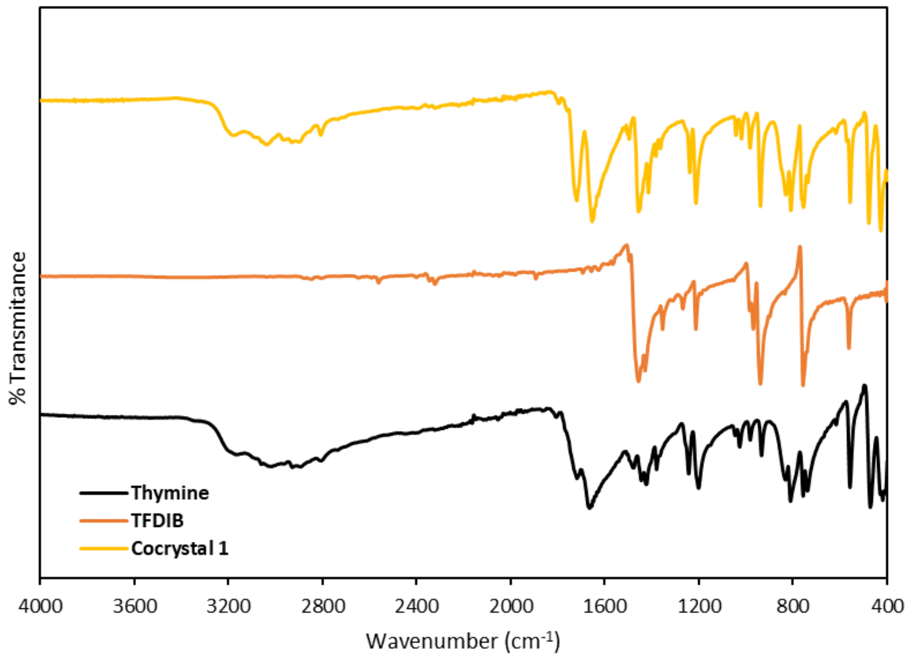

Figure S6. Comparison of ATR-FT-IR spectra of 1-ethyluracil, 1,2,4,5-tetrafluoro-3,6-diiodobenzene (TFDIB) and cocrystal **2**.

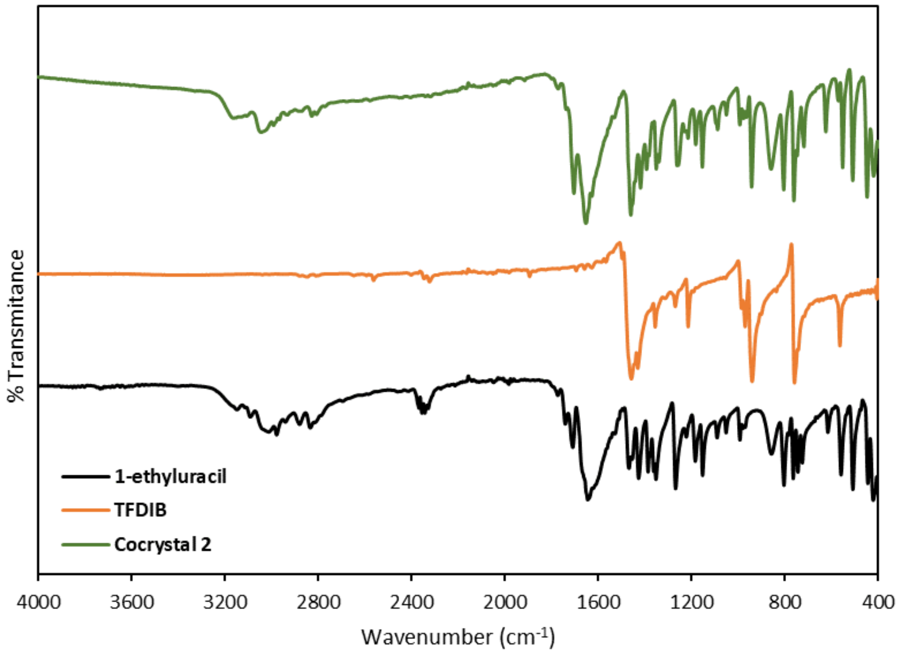

Figure S7. Comparison of ATR-FT-IR spectra of 5-fluorouracil, 1,2,4,5-tetrafluoro-3,6-diiodobenzene (TFDIB) and cocrystal **3**.

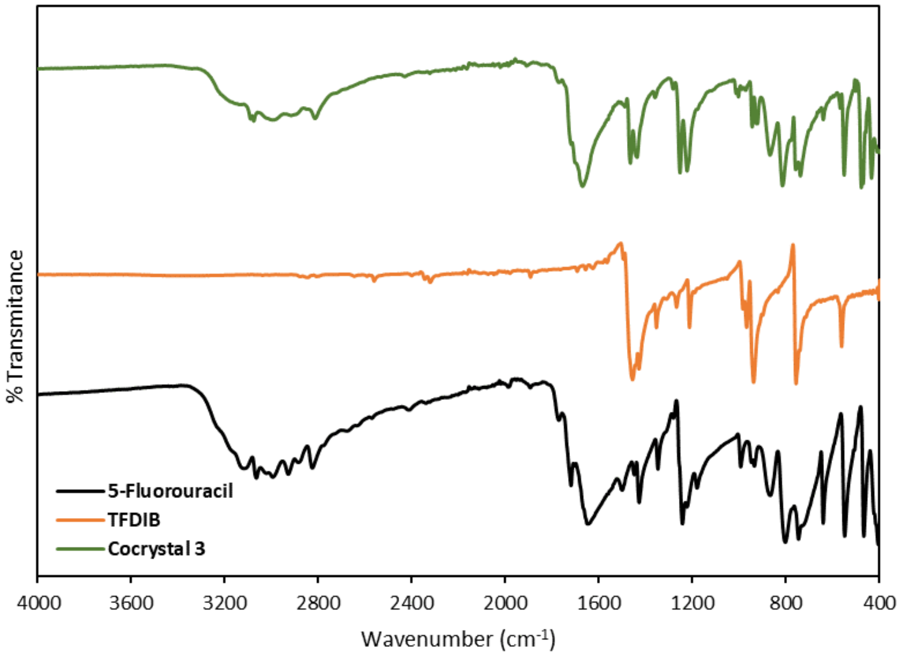

Table S2. Some characteristic frequencies for the modified uracil compounds, the coformer and the new cocrystals **1-3**.

	TFDIB	THY	1	1ETURA	2	5FU	3
**νC-C**	1455	-	1456	-	1458	-	1463
**νC-F**	937	-	940	-	937	-	943
**νC-I**	755	-	753	-	758	-	758
**νC=O**	-	1720/1667	1717/1654	1739/1708/1643	1704/1651	1720/1648	1672
**νN-H**	-	3166	3179	3147/3090	3166	3115	3150/3088

Figure S8. TGA-DSC of the cocrystal **1**.

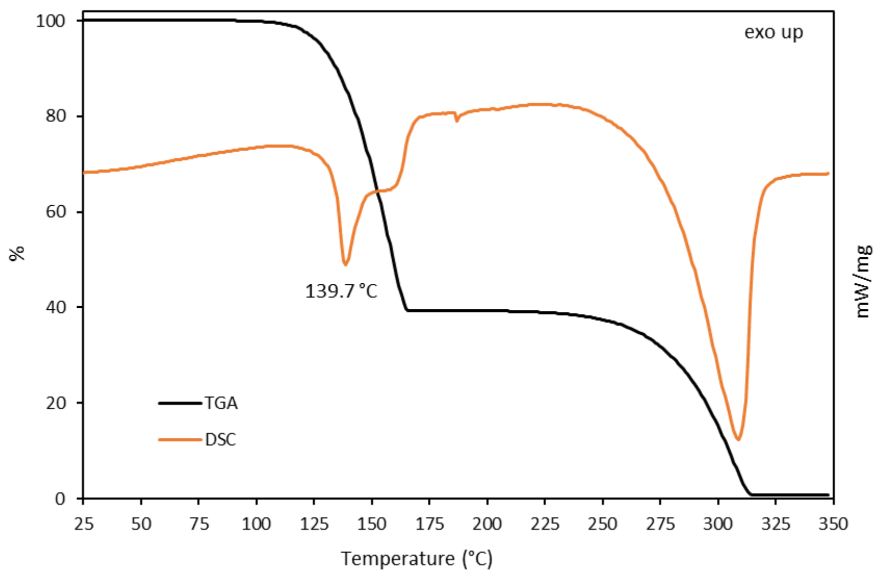

Figure S9. TGA-DSC of the cocrystal **2**.

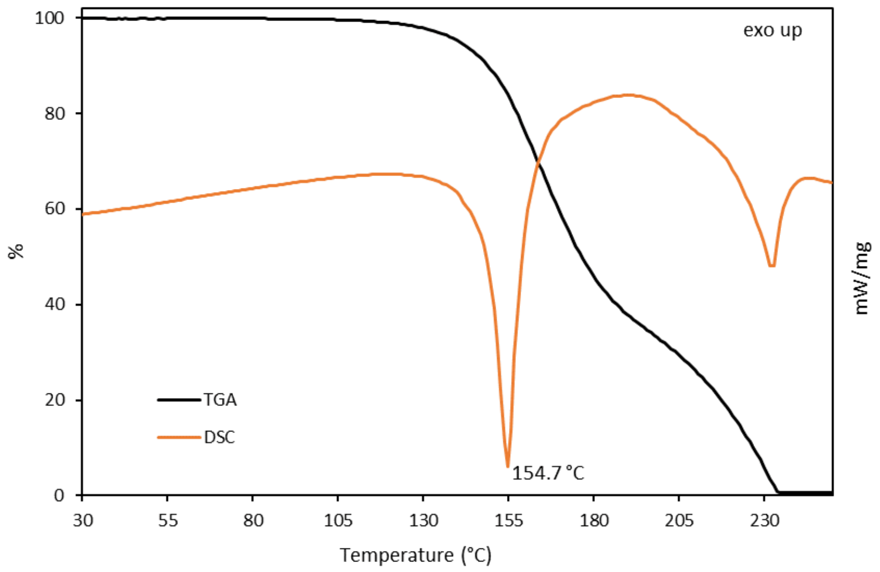

Figure S10. TGA-DSC of the cocrystal **3**.

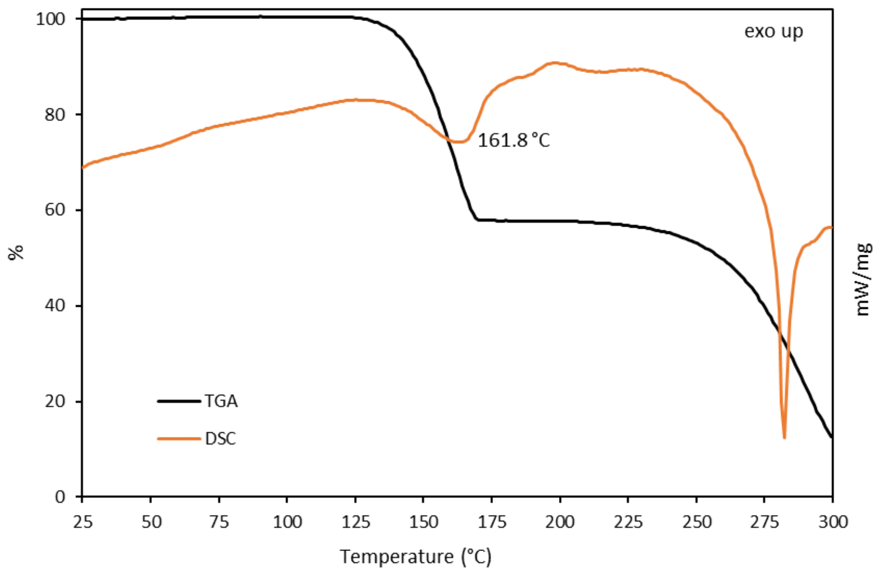

Figure S11. X-bonds angles in cocrystals **1**-**3**.

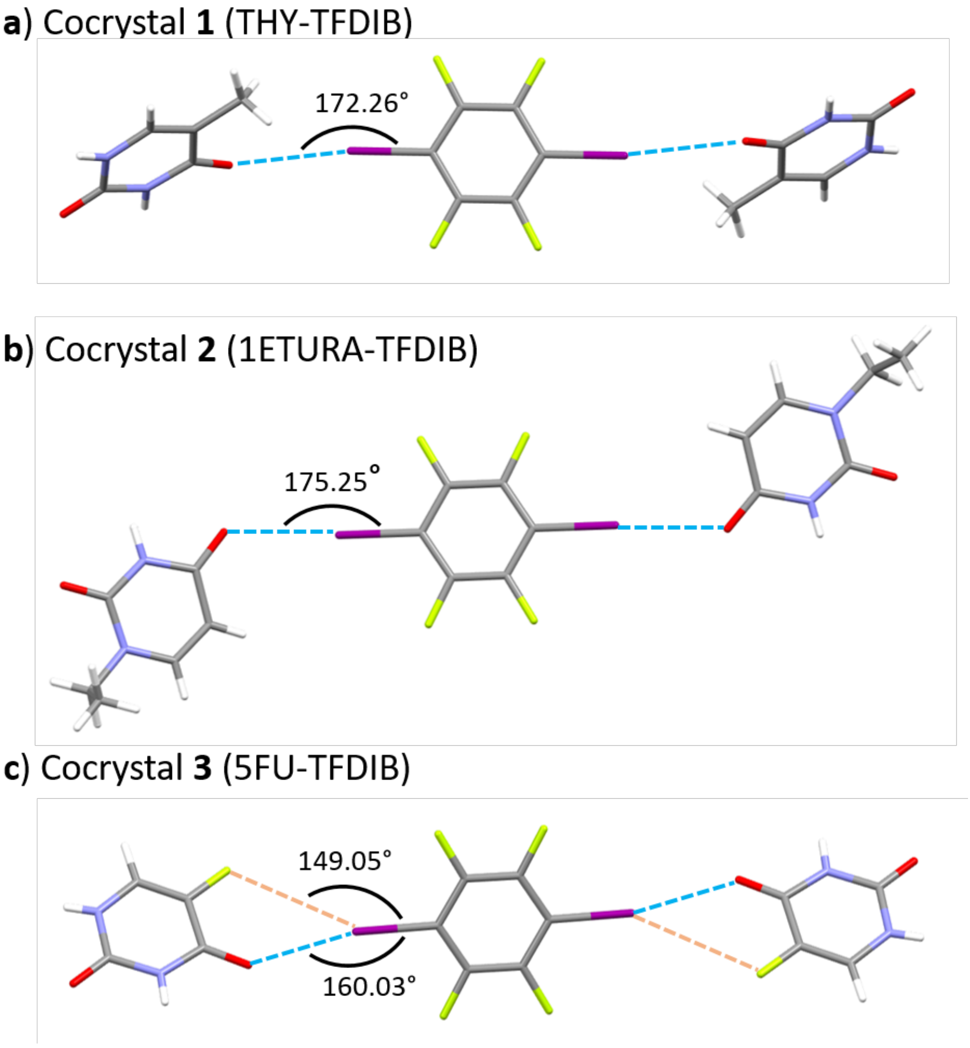

Table S3. Hydrogen bonds for cocrystals **1** (THY-TFDIB), **2** (1ETURA-TFDIB) and **3** (5FU-TFDIB) [Å and °].

Cocrystal	D-H...A	d(D-H)	d(H...A)	d(D...A)	<(DHA)
**1**	N(3)—H(3)···O(6) #1	0.86	2.06	2.920 (7)	173
	N(1)—H(1)···O(2) #2	0.86	1.99	2.850 (7)	174
**2**	N(3)-H(3)···O(2')	0.86	2.03	2.884(2)	172.7
	N(3')-H(3')···O(2)	0.86	2.02	2.864(2)	167.7
	C(7)-H(7A)···O(4) #3	0.97	2.51	3.335(3)	142.7
	C(7')-H(7'1)···O(4') #4	0.97	2.47	3.245(3)	137.2
**3**	N(1)—H(1)···O(12)	0.78 (4)	2.06 (4)	2.819 (4)	167 (4)
	N(3)—H(3)···O(12) #5	0.78 (4)	2.03 (4)	2.805 (4)	174 (4)
	C(6)—H(6)···O(4) #6	1.01 (4)	2.41 (4)	3.360 (5)	157 (3)
	N(11)—H(11)···O(2) #6	0.82 (5)	2.02 (5)	2.847 (4)	177 (5)
	N(13)—H(13)···O(2)	0.82 (5)	1.96 (5)	2.781 (4)	173 (5)
	C(16)—H(16)···F(1) #7	0.92 (4)	2.59 (4)	3.221 (5)	127 (3)
	C(16)—H(16)···O(14) #6	0.92 (4)	2.51 (4)	3.353 (5)	153 (3)

Symmetry transformations used to generate equivalent atoms:
#1 −*x*, −*y*+1, −*z*+2; #2 −*x*−1, −*y*+2, −*z*+2.

#3 x-1, y, z; #4 x+1,y,z

#5 *x*−1, *y*, *z*; #6 *x*+1, *y*, *z*; #7 −*x*+3/2, *y*−1/2, −*z*+3/2.
